# Failed reciprocity in core social roles and cardiovascular disease mortality: prospective results from the U.S. health and retirement study

**DOI:** 10.1186/s12889-025-22259-5

**Published:** 2025-03-21

**Authors:** Johannes Siegrist, Timothy A. Matthews, Jian Li

**Affiliations:** 1 Institute of Medical Sociology, Centre for Health and Society, Faculty of Medicine, University of Düsseldorf, Düsseldorf, Germany; 2 Department of Environmental & Occupational Health, College of Health & Human Development, California State University Northridge, Northridge, CA USA; 3 Departments of Environmental Health Sciences and Epidemiology, Fielding School of Public Health, School of Nursing, University of California Los Angeles, 650 Charles E. Young Drive South, Los Angeles, CA 90095 USA

**Keywords:** Cardiovascular disease, Combined psychosocial risks, Effort-reward imbalance, Mortality, Social reciprocity

## Abstract

**Background:**

Several epidemiologic cohort studies documented increased risks of cardiovascular disease (CVD) resulting from exposure to psychosocial stress at work, as measured by theoretical models, such as the demand-control model and effort-reward imbalance (ERI) model. Few studies extended these concepts beyond paid work. With this study, we explore associations of ERI in family and household work, voluntary work and/or partnership with prospective CVD mortality risk, in addition to ERI at work.

**Methods:**

In the frame of the U.S. Health and Retirement Study (HRS), 4,642 individuals with mean age 61.5 who were employed at baseline (2006–2008) provided full data on ERI at work and beyond work, sociodemographic factors, lifestyle behaviors, and health conditions. Mortality data through the end of 2018 were available through linkage to the National Death Index. Participants were dichotomized into ‘low’ and ‘high’ group for each ERI dimension. Prospective associations of the two types of ERI at baseline with CVD mortality during follow-up were estimated, using competing-risk Cox proportional hazards regression.

**Results:**

Independ effects of work ERI and social ERI on CVD mortality risk were observed (HR: 1.66 [1.08; 2.53] and HR: 1.56 [1.02; 2.38], respectively). The hazard ratio of CVD mortality among participants with joint exposure was 2.58 [1.49; 4.45], compared to unexposed participants. This risk was further augmented (HR: 3.38 [1.45; 7.85]) in participants with cardiometabolic disease at baseline.

**Conclusion:**

Failed reciprocity in core social roles of adult life is associated with increased CVD mortality risk in this study of older employed persons in the U.S.

**Supplementary Information:**

The online version contains supplementary material available at 10.1186/s12889-025-22259-5.

## Introduction

Reciprocity between ‘give’ and ‘take’ in effortful social exchange is a perennial evolutionary principle of sustainable human interaction, enabling trust and cooperation [1]. Violating this norm results in social conflicts and personal distress among those affected. This notion underlies a theoretical model of stressful psychosocial work, termed ‘effort-reward imbalance’ (ERI) [2]. With its focus on the employer-employee relationship, it posits that high efforts spent at work that are not met by appropriate rewards (earnings; promotion prospects and security; appreciation) result in distressing experiences with adverse long-term effects on health and well-being. As a complementary concept to another internationally established model of stressful psychosocial work, ‘demand-control’ (or ‘job strain’) [3], it has been examined in a variety of occupational health investigations, with particular emphasis on cardiovascular disease (CVD). Evidence from prospective cohort studies of these two work-stress models points to moderately elevated relative risks of incident coronary heart disease [4–7], of recurrent coronary events [8], and of cardiovascular or all-cause mortality [9, 10]. Both models are based on core psychobiological stress mechanisms activating the autonomic nervous system and the hypothalamic-pituitary-adrenal axis via corticolimbic pathways [11]. How recurrent stressful experience impairs the cardiovascular system has been documented in recent reviews [12, 13].

Given the fact that the rather general notion of failed reciprocity in social exchange has been limited to the work role, one may ask whether similar health effects can be expected from effortful contributions in the frame of other core social roles of adult life, dealing with obligations and expectations in partnership, in family and household work, or in voluntary work. Although this question was studied with regard to selected mental health outcomes [14–16], no study so far examined effects on physical diseases, including mortality risk. Related to this lack of knowledge is the question whether a combined exposure to the two types of failed reciprocity, at work and in a further social role of adult life, magnifies the associated health risk. In line with research on the stress accumulation hypothesis [17], a substantially elevated health risk is expected from combined exposure, compared with single and no exposure.

With this study, we set out to examine these two questions in relation to a health outcome with high relevance, CVD mortality. By constructing two summary measures of reciprocal exchange at work and in the other social roles mentioned, independent and joint effects of ERI on risk of CVD mortality are estimated. In the frame of a prospective cohort study of older employees in the United States (U.S.), we linked exposure data collected in 2006–2008 with reports of CVD mortality documented up to 2018, adjusting for important covariate variables (see Methods). The following two hypotheses are examined. First, ERI in other social roles and ERI at work are independently associated with an elevated risk of CVD mortality. Second, this mortality risk is substantially increased among participants who are simultaneously exposed to both stressful exposures. This magnified effect may be due to a generalized, aggravated state of social reward frustration that threatens the persons’ self-esteem by recurrent failure of experiencing just exchange, a basic human need. As a supplementary research question, we explore whether the effects proposed by the second hypothesis are particularly pronounced among participants with signs of cardiometabolic disease at study entry. Given an advanced stage of vulnerability, their capability of successfully coping with stressful encounters may be compromised, thus increasing their risk of a fatal event. In a landmark investigation on the prospective mortality risk of psychosocial stress at work, as measured by the demand-control model, this risk was significantly higher in the subgroup of participants with cardiometabolic disease at entry, compared to the risk of participants without this disorder [10].

## Methods

### Study subjects

The Health and Retirement Study (HRS) is an ongoing, large, national, population-based longitudinal study among U.S. adults aged 50 + since 1992, with publicly accessible datasets for scientific research [18]. Our analyses were focused on individuals with employment who provided information on psychosocial experiences in 2006 and 2008, the baseline time-point for this epidemiological investigation. These two waves were combined to retrieve full data on target variables, as survey items corresponding to psychosocial stressors were administered to roughly half of the study sample in 2006 and 2008, respectively. The HRS had a total sample size of 43,399 participants in 2006–2008, and 7,265 were currently working. Mortality data for the HRS cohort were available through 2018, representing a maximum follow-up period of 12 years. The size of the final analytic sample was 4,642 after excluding individuals with missing values on psychosocial information and covariates (the process of sample size selection for our study using the HRS data is shown in Supplementary Fig. 1). This significant sample loss was mainly due to the fact that the questionnaire assessing psychosocial items was administered to roughly half the study sample in 2006 and 2008, respectively. Non-participation at each wave thus increased the number of missing values. We compared the primary sociodemographic characteristics of the study sample with the non-responding sample via Chi-square test. Participants who were male, Black, or who had lower education or income levels were more likely to have missing ERI data.

### Measures

Both work ERI and social ERI were measured at baseline, via the self-administered Psychosocial Leave-Behind questionnaire, which was given to participants who completed in-person interview. The overall response rates for the HRS in-person interview were 89%, and 74% for Psychosocial Leave-Behind questionnaire during 2006–2008 [18]. A standardized work ERI instrument consisting of two dimensions, Effort and Reward, was applied in the HRS study, with responses on a 4-point Likert scale. Four items measured effort, (1) “My job is physically demanding,” (2) “I am under constant time pressure due to a heavy workload,” (3) “Considering the things I have to do at work, I have to work very fast,” and (4) “In my work I am free from conflicting demands that others make.” Five items measured reward, (1) “I receive the recognition I deserve for my work,” (2) “My salary is adequate,” (3) “My job promotion prospects are poor,” (4) “My job security is poor,” and (5) “I receive adequate support in difficult situations.” For both, a sum score of items was calculated, then effort/reward ratio was obtained by dividing effort by reward, weighted by the number of items in each dimension, where higher values reflected higher levels of work stress [19, 20]. The work effort/reward ratio was dichotomized by the median split point into two low and high groups.

Social ERI was measured with 3 items, including (1) “I have always been satisfied with the balance between what I have given my partner and what I have received in return,” (partnership role), (2) “I have always received adequate appreciation for providing help in my family,” (family and housework role), (3) “In my current major activity I have always been satisfied with the rewards I received for my effort” (voluntary work role). A 5-point Likert scale was used to record these responses. The sum score was calculated, with higher values indicated higher stress levels [21]. Similarly, social ERI was also dichotomized by the median split point into two low and high groups.

At baseline, information on sociodemographic factors, lifestyle behaviors, and health conditions was collected through in-person interviews. The following covariates were selected because they are known as traditional risk factors for CVD among workers [22] which have commonly been considered when conducting research on occupational cardiology in previous HRS reports [23]: age, sex (male, female), marital status (married, not married), race (White, Black, others), educational attainment (high school or less, some college or more), annual household income (<$72,000, ≥$72,000 USD by median point), current cigarette smoking (no, yes), alcohol consumption (low to moderate drinking, heavy drinking [more than 2 drinks per day for male and 1 drink per day for female]), physical exercise (low, high [more than once a week vigorous or moderate physical exercise]), body mass index (BMI, calculated as weight in kilograms divided by height in meters squared; < 25.0, 25.0-29.9 [overweight], ≥ 30.0 [obese]), hypertension (no, yes), diabetes (no, yes), any type of CVD, including stroke, heart attack/myocardial infarction, coronary heart disease, angina, congestive heart failure, or other heart problems (no, yes).

The dependent variable and target outcome of interest was CVD mortality during the follow-up. In this study, CVD mortality data up to the end of 2018 were available through linkage to the National Death Index, and additionally through biennial exit interviews with surviving household members, with CVD mortality cases defined by ICD-10 codes I00-I99, together with timing of death [24].

### Statistical analysis

First, descriptive statistics were generated for baseline characteristics of the study subjects. Means and standard deviations (SDs) were calculated for continuous variables, and relative frequencies were examined for categorical variables, according to the levels of work ERI and social ERI. Statistical differences in covariates between ERI groups were determined by t-test for continuous variables, or by χ^2^ test for categorical variables. Second, prospective associations of work ERI and social ERI at baseline with time-to-event outcome, i.e., CVD mortality during follow-up, were estimated using competing-risk Cox proportional hazards regression, and non-CVD mortality was evaluated as a competing risk [25]. Estimates were calculated as hazard ratios (HRs) with 95% confidence intervals (CIs) and adjusted for covariates in 4 multivariable models. Model I was adjusted for age and sex. Further adjustment for marital status, race, education, and household income was added in Model II. Model III and Model IV were additionally adjusted for lifestyle behaviors (including smoking, alcohol drinking, and physical exercise) and health conditions (BMI, hypertension, diabetes, and CVD), respectively. Hypothesis tests were 2-sided at the 5% α level. Calendar time was used as the time scale of the Cox proportional hazards models for calculating length of follow-up in terms of person-years, and participants were right-censored at the end of follow-up (up to the end of 2018) or the time of deaths, depending on whichever came first. The proportional hazards assumptions of the Cox models were verified by the SAS PHREG procedure and ASSESS function with the PH option (*p* > 0.10). Independent associations of work ERI and social ERI with CVD mortality were estimated with mutual adjustment for two types of ERI. Joint associations of work ERI and social ERI with CVD mortality were estimated by creating a composite variable with different combinations of the two types of ERI (i.e., low work ERI, low social ERI [HR_00_ reference]; low work ERI, high social ERI [HR_10_]; high work ERI, low social ERI [HR_01_]; high work ERI, high social ERI [HR_11_]). Third, the relative excess risk due to interaction (RERI, = HR_11_– HR_10_– HR_01_ + 1) was calculated. An RERI greater than 0 indicates a positive interaction, equal to 0 indicates an additive interaction, and less than 0 indicates a negative interaction; its 95% CI was computed according to Andersson methods [26]. The multiplicative interaction term of work ERI and social ERI with CVD mortality was also examined. Fourth, stratified analyses were further performed by the pre-existing cardiometabolic disease (any type of CVD or diabetes) at baseline, in line with previous research [10]. Finally, in order to test the robustness of the associations, we conducted sensitivity analyses: (i) with single effort and reward at work, as well as continuous measures of all ERI indicators (increase per SD) as independent variables in the regression analyses; (ii) additional adjustment for job control which is the core dimension of the job strain model, for testing independent effects of ERI on CVD mortality; (iii) for minimizing potential reverse causation, we also excluded CVD death cases that took place in the first 3 years of follow-up from the analyses; (iv) a more traditional cut-off point of 1 was used to dichotomize the work effort/reward ratio into two low and high groups. All analyses were conducted using the SAS 9.4 software package (SAS Institute, Cary, NC).

## Results

Main descriptive characteristics of the study subjects are given in Table 1. With a mean age of 61.5 years, the sample represented a generally sex-balanced working sample. More than 80% of participants were White, and a high proportion (> 60%) of participants had a low educational level (i.e., high school or less). In terms of behavioral and health-related features, only low proportions were cigarette smokers or heavy alcohol consumers. Yet, the prevalences of main cardiometabolic risk factors as well as cardiovascular diseases were remarkably high. Assessment of differences between ERI groups revealed that being younger, other race, low education, low income, smoking, and low levels of physical exercise were more likely to be associated with high work ERI; while being younger, female, White, and high education were more likely to be associated with high social ERI.

**Table 1:** Baseline characteristics of study subjects, N (%)

Characteristics	Overall (*n* = 4,642)	Work ERI		*p*-value	Social ERI		*p*-value
		Low (*n* = 2,458)	High (*n* = 2,184)		Low (*n* = 2,521)	High (*n* = 2,121)	
Age, mean ± SD (yr)	61.49 ± 7.14	62.76 ± 7.35	60.07 ± 6.62	< 0.001	62.56 ± 7.39	60.23 ± 6.61	< 0.001
Sex				0.73			< 0.001
Male	2,262 (48.73)	1,192 (48.49)	1,070 (48.99)		1,311 (52.00)	9,51 (44.84)	
Female	2,380 (51.27)	1,266 (51.51)	1,114 (51.01)		1,210 (48.00)	1,170 (55.16)	
Marital status				0.49			0.29
Married	3,086 (66.48)	1,623 (66.03)	1,463 (66.99)		1,693 (67.16)	1,393 (65.68)	
Not married	1,556 (33.52)	835 (33.97)	721 (33.01)		828 (32.84)	728 (34.32)	
Race				0.02			0.02
White	3,785 (81.54)	2,035 (82.79)	1,750 (80.13)		2,026 (80.37)	1,759 (82.93)	
Black	529 (11.40)	273 (11.11)	256 (11.72)		318 (12.61)	211 (9.95)	
Others	328 (7.06)	150 (6.10)	178 (8.15)		177 (7.02)	151 (7.12)	
Educational attainment				0.02			< 0.001
Low (high school or less)	2,877 (61.98)	1,485 (60.41)	1,392 (63.74)		1,619 (64.22)	1,258 (59.31)	
High (some college or more)	1,765 (38.02)	973 (39.59)	792 (36.26)		902 (35.78)	863 (40.69)	
Household income (annual U.S. dollars)				0.02			0.45
Low (≤ 72,000)	2,304 (49.63)	1,179 (47.97)	1,125 (51.51)		1,264 (50.14)	1,040 (49.03)	
High (> 72,000)	2,338 (50.37)	1,279 (52.03)	1,059 (48.49)		1,257 (49.86)	1,081 (50.97)	
Smoking status				< 0.001			0.14
No	3,948 (85.05)	2,137 (86.94)	1,811 (82.92)		2,162 (85.76)	1,786 (84.21)	
Yes	694 (14.95)	321 (13.06)	373 (17.08)		359 (14.24)	335 (15.79)	
Alcohol consumption				0.63			0.97
Low or moderate drinking	4,474 (96.38)	2,366 (96.26)	2,108 (96.52)		2,430 (96.39)	2,044 (96.37)	
Heavy drinking	168 (3.62)	92 (3.74)	76 (3.48)		91 (3.91)	77 (3.63)	
Physical exercise				< 0.001			0.27
Low	2,032 (43.77)	1,014 (41.25)	1,018 (46.61)		1,085 (43.04)	947 (44.65)	
High	2,610 (56.23)	1,444 (58.75)	1,166 (53.39)		1,436 (56.96)	1,174 (55.35)	
BMI				0.10			0.78
<25.0	729 (15.70)	411 (16.72)	318 (14.56)		404 (16.03)	325 (15.32)	
25.0-29.9	2,643 (56.94)	1,392 (56.63)	1,251 (57.28)		1,434 (56.88)	1,209 (57.00)	
≥30.0	1,270 (27.36)	655 (26.65)	615 (28.16)		683 (27.09)	587 (27.68)	
Hypertension				0.22			0.83
No	2,993 (64.48)	1,565 (63.67)	1,428 (65.38)		1,622 (64.34)	1,371 (64.64)	
Yes	1,649 (35.52)	893 (36.33)	756 (34.62)		899 (35.66)	750 (35.36)	
Diabetes				0.03			0.97
No	4,107 (88.47)	2,198 (89.42)	1,909 (87.41)		2,230 (88.46)	1,877 (88.50)	
Yes	535 (11.53)	260 (10.58)	275 (12.59)		291 (11.54)	244 (11.50)	
Cardiovascular disease				0.17			0.89
No	3,919 (84.42)	2,092 (85.11)	1,827 (83.65)		2,130 (84.49)	1,789 (84.35)	
Yes	723 (15.58)	366 (14.89)	357 (16.35)		391 (15.51)	332 (15.56)	

Two-sample t-tests were used to compare continuous variables; Chi-squared tests were used to compare categorical variables

Over the average follow-up length of 10.3 years, corresponding to 47,685 person-years; in total, 557 death cases (including 93 CVD death cases) were identified, out of 4,642 study participants. The overall CVD mortality rate was 1.95 per 1,000 person-years. The findings related to the first hypothesis are displayed in Table 2, where the cardiovascular mortality risk was estimated for the two concepts of failed social reciprocity in four models, adjusting for a number of confounders in a stepwise approach. Two observations were noticeable. First, the stepwise adjustment procedure did not substantially modify the risk of CVD mortality, with the exception of adjusting for sociodemographic factors (from Model I to Model II). Second, for work ERI and social ERI, a moderately elevated CVD mortality risk was observed by 66% and 56%, respectively.

**Table 2:** Independent associations of two types of ERI at baseline with risk of CVD mortality among U.S. Older employees

ERI	CVD mortality rate (per 1,000 person-years)	Model I HR (95% CI)	Model II HR (95% CI)	Model III HR (95% CI)	Model IV HR (95% CI)
Work ERI					
Low	1.69	1.00	1.00	1.00	1.00
High	2.23	2.79 (1.18, 2.72) **	1.68 (1.10, 2.56) *	1.69 (1.11, 2.58) *	1.66 (1.08, 2.53) *
Social ERI					
Low	1.87	1.00	1.00	1.00	1.00
High	2.04	1.50 (0.99, 2.28)	1.54 (1.02, 2.35) *	1.56 (1.02, 2.37) *	1.56 (1.02, 2.38) *

Model I: adjusted for age and sex at baseline

Model II: Model I + additionally adjusted for marital status, race, education, and household income at baseline

Model III: Model II + additionally adjusted for smoking, alcohol drinking, and physical exercise at baseline

Model IV: Model III + additionally adjusted for BMI, hypertension, diabetes, and CVD at baseline

* *p* < 0.05, ** *p* < 0.01

**Fig. 1:**
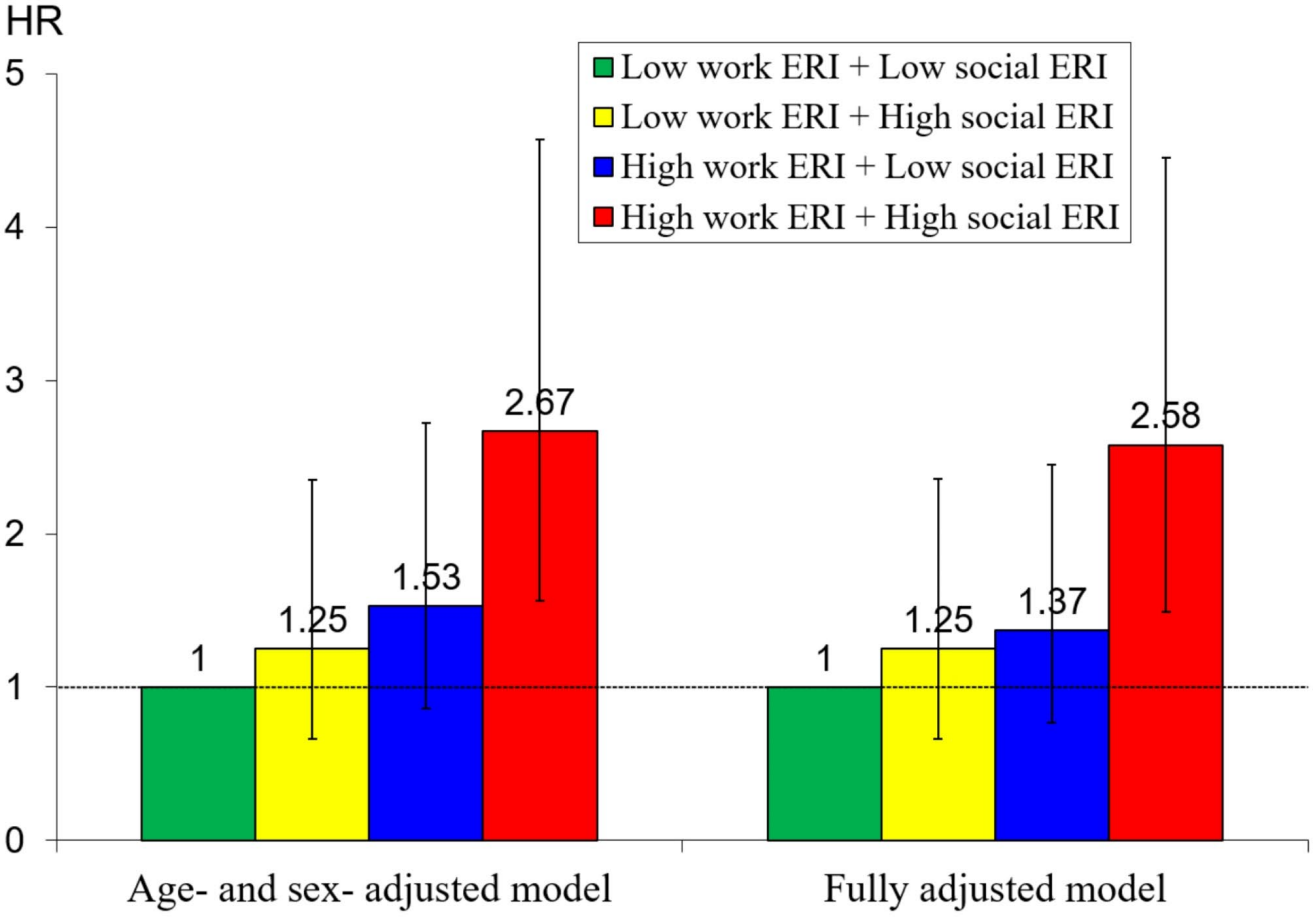
Joint associations of two types of ERI at baseline with risk of CVD mortality among U.S. older employees 95% CIs are presented with error bars Fully adjusted model includes age, sex, marital status, race, education, household income, smoking, alcohol drinking, physical exercise, BMI, hypertension, diabetes, and CVD at baseline RERI = 0.95 (95% CI: -0.35, 2.25) in the fully adjusted model

The second hypothesis was examined by constructing a combined variable of the two concepts of failed social reciprocity, with four categories. As illustrated in Fig. 1, compared to participants who were free from both types of stressful experience, those with joint exposure had a more than two-fold increased CVD mortality risk. Again, stepwise adjustment of different confounders did not modify the final risk estimates (for details please see Supplementary Table 1). Furthermore, it is of interest to see that no other combination of joint exposures was associated with a significantly elevated CVD mortality risk. With regard to a potential interaction analysis, the RERI was not significantly different from 0 (in the fully adjusted model, point estimate = 0.95, with 95% CI = -0.35 to 2.25), indicating an additive interaction between work ERI and social ERI; the multiplicative interaction term of two types of ERI with CVD mortality was also not significant (fully adjusted *p* = 0.35).

As mentioned, we additionally explored whether the strength of effect differed between participants with cardiometabolic disease at study entry, compared to the effect among those without disease. Respective results are displayed in Table 3, documenting a more than three-fold elevated mortality risk following double exposure in this susceptible group, compared to a risk of 2.28 in the remaining sample of healthy participants.

**Table 3:** Prospective associations of two types of ERI at baseline with risk of CVD mortality among U.S. Older employees, stratified by cardiometabolic disease at baseline

ERI	Fully adjusted HR (95% CI)
Study participants without cardiometabolic disease at baseline (*n* = 3,385)	
Work ERI	
Low	1.00
High	1.48 (0.84, 2.60)
Social ERI	
Low	1.00
High	1.54 (0.88, 2.68)
Double ERI	
Low work ERI + Low social ERI	1.00
Low work ERI + High social ERI	1.60 (0.73, 3.50)
High work ERI + Low social ERI	1.54 (0.71, 3.32)
High work ERI + High social ERI	2.28 (1.08, 4.82) *
Study participants with cardiometabolic disease at baseline (*n* = 1,257)	
Work ERI	
Low	1.00
High	2.02 (1.04, 3.94) *
Social ERI	
Low	1.00
High	1.70 (0.88, 3.28)
Double ERI	
Low work ERI + Low social ERI	1.00
Low work ERI + High social ERI	0.76 (0.24, 2.38)
High work ERI + Low social ERI	1.17 (0.48, 2.83)
High work ERI + High social ERI	3.38 (1.45, 7.85) **

Adjusted for age, sex, marital status, race, education, household income, smoking, alcohol drinking, physical exercise, BMI, and hypertension baseline

* *p* < 0.05, ** *p* < 0.01

Several sensitivity analyses were performed to further examine these findings. First, as in case of the work role, the summary estimate is based on the combination of two scales. In Supplementary Table 2, independent effects of the scales ‘effort’ and ‘reward’ were additionally analysed. Moreover, findings with continuous measure of the predicting variables are also presented. In all cases, statistically significant associations are observed. Second, when additionally adjusting for job control in the multivariable regression analysis, given a high correlation coefficient between the ‘control’ and the ‘reward’ (correlation coefficient *r* = 0.62, *p* < 0.001), a reduced effect of work ERI on CVD mortality risk was expected and observed (HR reduction from 1.66 to 1.36). However, the effect of joint exposure on CVD mortality risk remained significant (Supplementary Table 3). Third, in an attempt to reduce bias due to reverse causation, we excluded CVD death cases occurring during the first three years. It is possible that individuals with advanced manifest disease display higher levels of stressful experiences. In Supplementary Table 4, it turned out that such exclusion did not substantially change the results. Using the cut-off point ‘1’ to dichotomize two levels of work ERI, the findings were nearly unchanged (Supplementary Table 5).

## Discussion

In this study, employed older individuals whose contributions to the social roles of partnership, family and household work, and voluntary work were not reciprocated exhibited an elevated CVD mortality risk, compared to those experiencing just exchange in these roles. To the best of our knowledge, this is the first time that an established theoretical concept of chronically stressful experience at work, ERI, has been extended to other core social roles in adult life, by linking it to a significant physical health outcome, i.e., CVD mortality. Furthermore, the study documented a substantially increased CVD mortality risk among those who were simultaneously exposed to both types of failed social reciprocity, at work and in other social roles, compared to those unexposed. These findings are of theoretical interest as they complement the dominant paradigm of stress research focusing on lack of control [27] by emphasizing the significance of lack of reward following expended effort [28]. The finding of an association of ERI at work with CVD mortality supports earlier evidence from Europe [9, 10], but prospective links of failed reciprocity in other social roles with health so far were not reported for physical health outcomes, but were restricted to mental health [15, 16]. Few studies reported effects of the combined effect of two psychosocial stressors on health outcomes, compared to single exposures. Exploring associations with outcomes relevant to CVD, results indicated either separate effects (e.g. on atherosclerotic progression [29]) or combined effects exceeding single estimates (e.g. on increase of ambulatory blood pressure [30] or on all-cause mortality risk [31]). However, in most cases, these other social roles were not measured in terms of the theoretical models mentioned.

Sensitivity analyses were performed to (1) examine continuous measures of ERI indicators due to the fact that analyses restricted to a binary predictor result in a substantial loss of information, (2) consider another important psychosocial work characteristic (i.e., ‘job control’ [3]), and (3) minimize the bias due to potential reverse causation (exclusion of early cases of CVD deaths). We did not address the statistical option of adjusting for separate effects of ‘effort’ and ‘reward’ when estimating the health effect of the effort-reward ratio [32]. The theoretical model does not imply a synergistic effect of the two scales, but rather requires a quantitative assessment of the (im)balance between the two scales at individual data level, given the proposed non-linear association with health outcomes [33].

In a biopsychosocial perspective of health and disease, our findings support the notion of a direct impact of a chronically stressful psychosocial environment on CVD development, triggered by sustained activation of the brain’s corticolimbic circuits. Preliminary evidence demonstrated a prospective association of enhanced *stress-related* amygdalar activation, with increased risk of cardiovascular disease [34], and experimental findings in animal models showed that positive stimulation of the brain reward system via dopaminergic neurons improves vascularization and cardiac performance following acute myocardial infarction [35]. In experimental human studies, violation of the principle of reciprocity in social exchange was associated with sustained activation of the ventromedial prefrontal cortex [36], and of the anterior cingulate and insular cortex [37, 38]. These observations suggest that distinct brain reward circuits are activated by the experience of disadvantageous social inequality.

## Strengths and limitations

The main strength of this study concerns the theoretical enrichment of research on chronic psychosocial stress and health, emphasizing a relevant role of experienced social reward deficiency in effortful roles of adult life within and beyond the world of work. CVD mortality is a significant health outcome in terms of population health, and the two-fold elevated risk observed is comparable to the contribution of several established behavioral risk factors to CVD mortality (e.g., smoking [39]). Another strength relates to the application of the competitive model of Cox regression analysis that takes the effect of predictors on other causes of mortality into account [25]. The specific effects on CVD mortality are in line with documented evidence on pathways between chronic psychosocial stress and development of cardiovascular pathology [12, 13]. Adjusting the proposed effects by including up to thirteen covariates into stepwise multivariable regression analysis is considered a further strength of this study.

Nevertheless, several limitations need to be mentioned. First, we could not include measured biomedical data on important cardiovascular risk factors, such as blood pressure, body weight, and serum glucose, as these were only available for subgroups of the HRS sample. A respective significant sample reduction would have prevented a reliable test of our research hypotheses. However, self-reported data on these risk factors were previously applied in exploring contribution of (un)employment to CVD in previous HRS research [23]. A second limitation concerns the measurement of failed reciprocity in the three non-work-related social roles. Although validated scales were available for unbalanced reciprocity in the partnership role [14] and in the role of family and household work [16], the current study protocol included only single items for each type of exchange. These proxy measures do not fully reflect the stressful aspects inherent in unjust exchange. Therefore, future studies need to address this limitation by applying validated scales. ERI at work was assessed by the short version of the two psychometrically validated scales ‘effort’ and ‘reward’ [19], with the exception that the reward scale included only five out of seven items. In this context, we should also consider that ERI data were restricted to the baseline assessment, thus preventing analysis of change over time. Previous reports indicate that accumulated exposure over time increases the consecutive health risk [40]; thus, it is possible that the observed risk in our study was underestimated. A third limitation concerns the sample loss. Although we explained the design-induced lack of data, the observed socioeconomic difference between the study sample and the non-responding sample (less ERI data among males, black, and socioeconomically deprived people) do not allow an extension of the study findings to the initial sample of working participants. Fourth, with a limited sample size, our study reported 93 cases of CVD deaths. With this restricted number of cases, extended subgroup analyses were not feasible. Several factors, such as sex, race, socioeconomic position, age, or branch of economic activity could have revealed differences in mortality risk that remain unnoticed. This argument, again, calls for a replication study based on more comprehensive measures and a larger sample size. Furthermore, with an employed population with average age over 60 years, it is probable that a ‘healthy worker effect’ contributes to an underestimation of the CVD mortality risk, as employees with disadvantageous, highly stressful working conditions are at high risk of disability-related early retirement [41]. Finally, we do not know to what extent the reported findings can be generalized beyond the sample of the HRS study, representing a selected, older population of employed people in one country, the U.S.

## Clinical and public health implications

In its scientific statement on social determinants of risks and outcomes for CVD, the American Heart Association declared that scientifically established socioenvironmental conditions with adverse effects on CVD morbidity and mortality need to be recognized, assessed, and taken into account in medical strategies of prevention and treatment [42]. Work stress in terms of the demand-control and effort-reward imbalance models were mentioned as examples of such conditions. The results of the current study confirm the relevance of stressful work for premature CVD mortality among older working population in the U.S., and they demonstrate that such adverse effects can also result from reward frustration in family work or voluntary work. Raising awareness of these new risk factors among health care professionals can contribute to increase recognition, treatment and prevention of manifest CVD. Clearly, from a public health perspective, this awareness needs to be complemented by programs of structural improvement of working and employment conditions at the level of companies and organisations.

## Conclusions

In the present study, unrewarded effort in work-related and other core social roles is prospectively associated with an elevated risk of CVD mortality in older workers. Being exposed to both types of (work- and non-work-related) unjust social exchange doubles the risk of CVD mortality. These newly documented psychosocial risks deserve consideration in future CVD prevention strategies.

## Electronic supplementary material

Below is the link to the electronic supplementary material.

Supplementary Material 1

## Data Availability

Data used in this study are publicly available at https://hrsdata.isr.umich.edu/data-products/rand (accessed on 6 January 2023). The program code and scripts for statistical packages used to conduct the research are available from the corresponding author upon request.

## Abbreviations

BMI: Body Mass Index
CVD: Cardiovascular disease
ERI: Effort-Reward Imbalance
HRS: Health and Retirement Study
RERI: Relative Excess Risk due to Interaction
