# Supplementary material for: Failed reciprocity in core social roles and cardiovascular disease mortality: prospective results from the U.S. health and retirement study
Source: BMC Public Health. 2025 Mar 21;25:1090. doi: 10.1186/s12889-025-22259-5 (PMC11929191; doi:10.1186/s12889-025-22259-5)
Supplement: Supplementary file 1 — Supplementary Material 1 [file 12889_2025_22259_MOESM1_ESM.docx]

**Supplementary material**

**Failed reciprocity in core social roles and cardiovascular disease mortality: prospective results from the U.S. Health and Retirement Study**

All participants in HRS 2006-2008:

43,399

Participants who were not working:

36,134

Working population in HRS 2006-2008:

7,265

Participants with missing data on work ERI and social ERI:

2,349

Working population in HRS 2006-2008 with complete data:

4,916

Participants with missing data on covariates:

274

Final sample size for analyses:

4,642

**Supplementary Figure 1** Flowchart of sample size selection.

**Supplementary Table 1** Joint associations of two types of ERI at baseline with risk of CVD mortality among U.S. older employees

| ERI | CVD mortality rate (per 1,000 person-years) | Model I  HR (95% CI) | Model II  HR (95% CI) | Model III  HR (95% CI) | Model IV  HR (95% CI) |
| --- | --- | --- | --- | --- | --- |
| Double ERI |  |  |  |  |  |
| Low work ERI +  Low social ERI | 1.72 | 1.00 | 1.00 | 1.00 | 1.00 |
| Low work ERI +  High social ERI | 1.65 | 1.25 (0.66, 2.35) | 1.23 (0.65, 2.32) | 1.24 (0.66, 2.35) | 1.25 (0.66, 2.36) |
| High work ERI +  Low social ERI | 2.12 | 1.53 (0.86, 2.72) | 1.39 (0.78, 2.47) | 1.40 (0.79, 2.50) | 1.37 (0.77, 2.45) |
| High work ERI +  High social ERI | 2.32 | 2.67 (1.56, 4.57) ** | 2.58 (1.50, 4.42) ** | 2.63 (1.52, 4.54) ** | 2.58 (1.49, 4.45) ** |

Model I: adjusted for age and sex at baseline.

Model II: Model I + additionally adjusted for marital status, race, education, and household income at baseline.

Model III: Model II + additionally adjusted for smoking, alcohol drinking, and physical exercise at baseline.

Model IV: Model III + additionally adjusted for BMI, hypertension, diabetes, and CVD at baseline.

* p < 0.05, ** p < 0.01

**Supplementary Table 2** Associations of single ERI components at baseline with risk of CVD mortality among U.S. older employees

| ERI | Fully adjusted HR (95% CI) |
| --- | --- |
| Work Effort: Low | 1.00 |
| Hight | 1.53 (1.01, 2.33) * |
| Work Reward: Low | 1.00 |
| High | 0.61 (0.40, 0.93) * |
| Work Effort (continuous, increase per SD) | 1.26 (1.01, 1.58) * |
| Work Reward (continuous, increase per SD) | 0.78 (0.63, 0.96) * |
| Work ERI (continuous, increase per SD) | 1.34 (1.07, 1.68) * |
| Social ERI (continuous, increase per SD) | 1.27 (1.02, 1.58) * |

Adjusted for age, sex, marital status, race, education, household income, smoking, alcohol drinking, physical exercise, BMI, hypertension, diabetes, and CVD at baseline.

* p < 0.05, ** p < 0.01

**Supplementary Table 3** Prospective associations of two types of ERI at baseline with risk of CVD mortality among U.S. older employees, additionally adjusted for job control

| ERI | Model IV  HR (95% CI) | Model V  HR (95% CI) |
| --- | --- | --- |
| Work ERI |  |  |
| Low | 1.00 | 1.00 |
| High | 1.66 (1.08, 2.53) * | 1.36 (0.86, 2.15) |
| Social ERI |  |  |
| Low | 1.00 | 1.00 |
| High | 1.56 (1.02, 2.38) * | 1.48 (0.97, 2.27) |
| Double ERI |  |  |
| Low work ERI + Low social ERI | 1.00 | 1.00 |
| Low work ERI + High social ERI | 1.25 (0.66, 2.36) | 1.20 (0.64, 2.28) |
| High work ERI + Low social ERI | 1.37 (0.77, 2.45) | 1.15 (0.63, 2.09) |
| High work ERI + High social ERI | 2.58 (1.49, 4.45) ** | 2.02 (1.12, 3.65) * |

Model IV: adjusted for age, sex, marital status, race, education, household income, smoking, alcohol drinking, physical exercise, BMI, hypertension, diabetes, and CVD at baseline.

Model V: Model IV + additionally adjusted for job control at baseline.

* p < 0.05, ** p < 0.01

**Supplementary Table 4** Prospective associations of two types of ERI at baseline with risk of CVD mortality among U.S. older employees, with exclusion of 26 CVD death cases within the first 3 years

| ERI | Fully adjusted HR (95% CI) |
| --- | --- |
| Work ERI |  |
| Low | 1.00 |
| High | 1.64 (0.99, 2.71) |
| Social ERI |  |
| Low | 1.00 |
| High | 1.68 (1.02, 2.77) * |
| Double ERI |  |
| Low work ERI + Low social ERI | 1.00 |
| Low work ERI + High social ERI | 1.51 (0.73, 3.14) |
| High work ERI + Low social ERI | 1.49 (0.75, 2,98) |
| High work ERI + High social ERI | 2.76 (1.44, 5.28) ** |

Adjusted for age, sex, marital status, race, education, household income, smoking, alcohol drinking, physical exercise, BMI, hypertension, diabetes, and CVD at baseline.

* p < 0.05, ** p < 0.01

**Supplementary Table 5** Prospective associations of two types of ERI at baseline with risk of CVD mortality among U.S. older employees, with cut-off point ‘1’ to dichotomize two levels of work ERI

| ERI | Fully adjusted HR (95% CI) |
| --- | --- |
| Work ERI |  |
| Low | 1.00 |
| High | 1.64 (1.02, 2.65) * |
| Social ERI |  |
| Low | 1.00 |
| High | 1.56 (1.02, 2.38) * |
| Double ERI |  |
| Low work ERI + Low social ERI | 1.00 |
| Low work ERI + High social ERI | 1.50 (0.91, 2.45) |
| High work ERI + Low social ERI | 1.50 (0.72, 3.15) |
| High work ERI + High social ERI | 2.62 (1.43, 4.81) ** |

Adjusted for age, sex, marital status, race, education, household income, smoking, alcohol drinking, physical exercise, BMI, hypertension, diabetes, and CVD at baseline.

* p < 0.05, ** p < 0.01
